# Clinical significance of circulating FcγRIIa in the prognosis of patients with metastatic non-small cell lung cancer

**DOI:** 10.1007/s00432-026-06505-w

**Published:** 2026-05-24

**Authors:** Xiaojie Wang, Yu Teng, Mei Jiang, Xiaoting Zhao, Xiaokui Yang, Wentao Yue

**Affiliations:** 1 Department of Human Reproductive Medicine, Beijing Obstetrics and Gynecology Hospital, Capital Medical University. Beijing Maternal and Child Health Care Hospital., 251#, Yaojiayuan Road, Chaoyang, Beijing, 100026 China; 2 Department of Central Laboratory, Beijing Obstetrics and Gynecology Hospital, Capital Medical University. Beijing Maternal and Child Health Care Hospital., 251#, Yaojiayuan Road, Chaoyang, Beijing, 100026 China

**Keywords:** FcγRIIa, Biomarker, Metastasis, Non-small cell lung cancer, Prognosis

## Abstract

**Background:**

The tumor microenvironment plays a crucial role in determining the prognosis of tumors. Fc gamma receptor IIa (FcγRIIa), one of the three subtypes of FcγRII, is expressed on platelets and immunocytes such as macrophages and neutrophils. These cellular elements collectively contribute to the tumor microenvironment. Previous research has indicated that FcγRIIa activates platelets and inflammatory cells, thus participating in tumor growth and metastasis. Nonetheless, limited information is available regarding FcγRIIa levels in most cancer types. This study aimed to detect serum FcγRIIa in 333 patients with non-small cell lung cancer (NSCLC) and 100 healthy individuals, and to explore the relationship between serum FcγRIIa levels and clinical outcomes in patients with NSCLC.

**Methods:**

Serum samples from 333 patients with stage I–IV NSCLC and 100 healthy volunteers were analyzed using ELISA. The clinical and laboratory data underwent statistical analysis.

**Results:**

Circulating FcγRIIa levels were markedly increased in patients with NSCLC, especially in advanced pathologic stages.ROC curve analysis yielded an AUC of 0.7713 (95% CI: 0.7132–0.8264), with an optimal cutoff value of 2115.88 pg/mL based on the Youden index (sensitivity: 60.06%, specificity: 86.00%). Notably, 13% of healthy controls showed FcγRIIa levels above the cutoff, suggesting that elevated FcγRIIa may partially reflect systemic inflammatory status. Survival analysis in 333 patients with NSCLC showed markedly shorter overall survival in FcγRIIa-positive cases. Serum FcγRIIa levels were further identified to be significantly associated with metastatic status.

**Conclusion:**

This study demonstrated that circulating FcγRIIa levels rise along with tumor progression and may serve as a potential complementary prognostic indicator in metastatic NSCLC, though these findings require validation in prospective cohorts with comprehensive adjustment for inflammatory markers and treatment regimens.

## Introduction

NSCLC is a major contributor to cancer-related deaths worldwide, with NSCLC being the most common type (Stravopodis et al. 2023). The incidence of NSCLC has notably increased in China, especially among younger people. Moreover, with nearly 33% of the global population, NSCLC death in China accounts for more than 23% of that around the world, posing significant burdens (Li et al. 2022).

Human Fc gamma receptors (FcγRs) have long been elucidated as a family of heterogeneous molecules present on immune cells. They are three classes of FcγRs: FcγRI (CD64), FcγRII (CD32), and FcγRIII (CD16) (Gasparoto et al. 2021; Lu et al. 2019). These receptors bind to the constant fragment (Fc) of immunoglobulin G (IgG) antibodies and regulate the effector functions of IgG (Wang and Ravetch 2019). Allergy and autoimmune inflammation are closely connect to the triggering of mast cells and basophils by antibodies that specially recognize allergens and autoantigens (Jonsson et al. 2012). Both cell types possess receptors on their surface that specially bind to the Fc region of antibodies. The binding of antigen–antibody complexes to these receptors is crucial in regulating cell reactions (Cassard et al. 2012). Aggregation of high-affinity IgE receptors (FcεRs) and low-affinity IgG receptors (FcγRs) on the cell membrane triggers cells to discharge and release proinflammatory mediators (Willcocks et al. 2009) such as chemokines and cytokines. These mediators contribute to the clinical manifestations of allergy and autoimmune inflammation (Malbec et al. 2016; Pandey et al. 2013). Additionally, these receptors play a role in other biological reactions like antibody-dependent cellular cytotoxicity (ADCC) (Karlsson et al. 2018), endocytosis, phagocytosis, secretion of proinflammatory substances, and enhancement of antigen presentation. It is important to note that FcγRs vary in their specificity and affinity for different subclasses of IgG antibodies (Bruhns et al. 2009). Antigen-presenting cells employ FcγR-mediated endocytosis of immune complexes (ICs) and phagocytosis of tumor cells coated with antibodies as productive mechanisms for processing and presenting tumor antigens, leading to targeted T-cell immune responses against tumors (Clynes 2006; Arlen et al. 2010). In various cancer types, IgG antibodies are generated to identify cancer cells, form ICs, and activate FcγR (Chen et al. Jul 2012). When tumor-specific antibodies are produced, they can bind to FcγR and initiate cell-mediated anti-tumor immune responses (Nie et al. 2022).While FcγR-mediated immune responses contribute to anti-tumor defense (Nie et al. 2022), emerging evidence suggests that FcγRIIa activation may also promote tumor progression through platelet activation and inflammatory signaling,and establishment of a pro-tumorigenic microenvironment (Mitrugno et al. 2014). FcγRII, a form of FcγRs for monomeric IgG but avid for IgG-containing ICs, is dominantly expressed on myeloid cells, including granulocytes, monocytes, macrophages, platelets, and others (Braga et al. 2005; Jungi et al. 1988). FcγRIIa, as one of the activating receptors, triggers signaling through intracellular immunoreceptor tyrosine-based triggering domain, contributing to relevant inflammatory and immune responses (Alemán et al. 2021). In addition to its function in anti-tumor immunity, FcγRIIa was identified to be crucial in prostate cancer cell-triggered platelet activation. During the course, cancer cells directly stimulate platelet secretion and aggregation via platelet FcγRIIa, facilitating crosstalk between platelets and cancer cells (Mitrugno et al. 2014). Furthermore, platelet-secreted growth factors, known as platelet microparticles, have been shown to significantly enhance platelet aggregation, tumor growth and vascularization, and even the process of metastatic (Goubran et al. 2015; Labelle et al. 2011), all of which contribute to tumor progression. 

However, the involvement of FcγRIIa in NSCLC, especially in terms of serology, still lacks clarity. The research intended to detect the serum levels of FcγRIIa in 333 patients diagnosed with NSCLC and compare them with 100 age-matched healthy individuals. The objective was to explore the potential connection between the levels of serum FcγRIIa and the clinical outcomes of individuals with NSCLC.

## Materials and methods

### Patients and samples

During the time period spanning from January 2011 to December 2015, staff at the Sample Bank of Beijing Chest Hospital (Beijing, China) collected serum samples from 333 individuals diagnosed with NSCLC and 100 healthy volunteers. The detailed data was presented (Table 1). A group of 333 qualified patients were followed up for a maximum of 36 months after their diagnosis and were included in the survival analysis. Patients who had accepted preoperative adjunctive treatment upon enrollment were excluded. Blood samples were obtained from the individuals during their diagnosis and preserved at − 80 °C.

**Table 1:** Associations between the clinicopathologic characteristics and serum FcγRIIa levels

Variables	No. of patients	Positive	Negative	*P* value
Total number of patients	333	200 (60.1%)	133 (39.9%)	
Age				0.013^*^
≤ 60	165	88 (53.3%)	77 (46.7%)	
> 60	168	112 (66.7%)	56 (33.3%)	
Sex				0.690
Male	249	148 (59.4%)	101 (40.6%)	
Female	84	52 (61.9%)	32 (38.1%)	
Smoking status				0.831
Smoker	208	124 (59.6%)	84 (40.4%)	
Nonsmoker	125	76 (60.8%)	49 (39.2%)	
Pathologic type				0.638
A	170	100 (58.8%)	70 (41.2%)	
S	163	100 (61.3%)	63 (38.7%)	
pT status				< 0.001^***^
T1	51	21 (41.2%)	30 (58.8%)	
T2	150	85 (56.7%)	65 (43.3%)	
T3	52	35 (67.3%)	17 (32.7%)	
T4	80	59 (73.8%)	21 (26.3%)	
pN status				< 0.001^***^
N0	147	74 (50.3%)	73 (49.7%)	
N1	40	21 (52.5%)	19 (47.5%)	
N2	95	67 (70.5%)	28 (29.5%)	
N3	51	38 (74.5%)	13 (25.5%)	
pM status				< 0.001^***^
M0	239	130 (54.4%)	109 (45.6%)	
M1	94	70 (74.5%)	24 (25.5%)	
pTNM stage				< 0.001^***^
Ⅰ	75	32 (42.7%)	43 (57.3%)	
Ⅱ	75	40 (53.3%)	35 (46.7%)	
Ⅲ	90	57 (63.3%)	33 (36.7%)	
Ⅳ	93	72 (77.4%)	21 (22.6%)	

*P < 0.05, ^***^P < 0.001

*A* adenocarcinoma, *M* metastasis, *N* node, *S* squamous cell carcinoma, *T* tumor, *TNM* tumor-node-metastasis

#### Ethics approval and consent to participate

This study was approved by the Ethics Committee of Beijing Chest Hospital Affiliated to Capital Medical University and conducted in accordance with the Declaration of Helsinki. Written informed consent was obtained from all participants prior to their enrollment in this study.

### Enzyme-linked immunosorbent assay (ELISA)

We performed a quantitative sandwich ELISA (USCN Life Science, TX, USA) to measure the serum levels of FcγRIIa following the manufacturer's protocol. The diluted serum was added to the pre-coated microplate and incubated at 37 °C for 1 h. Any remaining solution was discarded, and diluted FcγRIIa detection antibodies were added to the plate and incubated at 37 °C for 1 h. After washing 3 times, the serum was incubated with the second antibody at 37 °C for 30 min. After washing 5 times, the substrate was added to each well and incubated at 37 °C for 15 min in the dark. Sulfuric acid was used to terminate the reaction. The absorption values were measured at 450 nm by a microplate reader. Each sample was independently performed in triplicate.

### Statistical analysis

We conducted the Kolmogorov–Smirnov test to examine the allocation of the data. The cutoff value of the serum level of FcγRIIa was determined using the receiver operating characteristic (ROC) curves. The serum level of FcγRIIa above the cutoff value were defined as “FcγRIIa positive”, while those below the cutoff value were considered “FcγRIIa negative”. We then used the chi-square test to investigate and categorize the clinical variables, exploring their associations with the serum level of FcγRIIa. Overall survival (OS) was estimated from the time of diagnosis until death related to cancer or the most recent follow-up. The Kaplan–Meier method and log-rank test were employed to examine the survival outcome. To determine the influence of different clinical variables on the prognosis of patients with NSCLC, a Cox proportional hazards model was applied. Additionally, a multifactorial analysis using stepwise regression was conducted to identify independent predictive variables. Stepwise regression was employed to identify the most parsimonious model in the context of limited subgroup sample sizes, following standard statistical practice for exploratory prognostic factor analysis. We acknowledge that this approach may introduce selection bias and that the resulting models require validation in independent cohorts before clinical application. SPSS 20.0 and GraphPad Prism 6 were employed, and *P* < 0.05 was considered statistically significant.

## Results

### Patients’ attributes

This research involved 333 individuals with NSCLC. The median age of these subjects was 60 years (range: 38–83 years), comprising 249 males (74.8%) and 84 females (25.2%). 163 patients (48.9%) had squamous cell carcinoma, and 170 (51.1%) had adenocarcinoma. In accordance with the TNM staging system, 75 individuals (22.5%) had stage I, 75 (22.5%) had stage II, 90 (27.1%) had stage III, and 93 (27.9%) had stage Ⅳ NSCLC.

### Increase in the levels of circulating FcγRIIa in NSCLC, especially in advanced pathologic stages

**Fig. 1:**
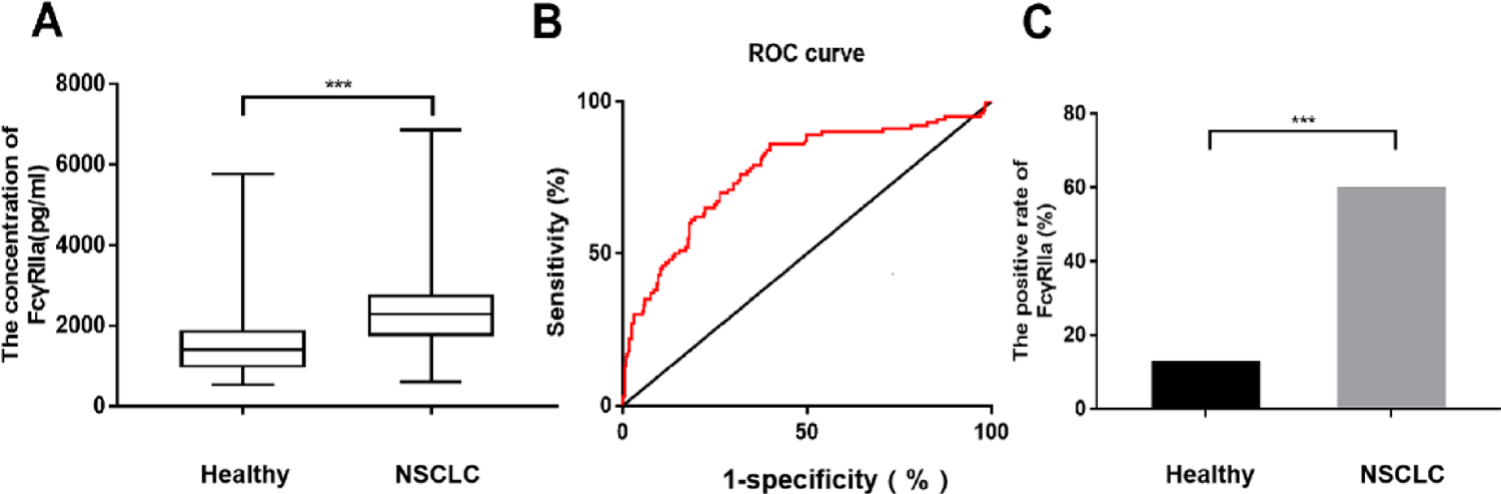
Serum FcγRIIa levels varied between patients with NSCLC and healthy controls. **A** Serum levels of FcγRIIa in NSCLC and healthy groups. **B** ROC curve analysis for serum FcγRIIa levels in distinguishing NSCLC patients from healthy controls. The AUC was 0.7713 (95% CI: 0.7132–0.8264), with an optimal cutoff value of 2115.88 pg/mL based on the Youden index (sensitivity: 60.06%, specificity: 86.00%). The arrow indicates the cutoff point on the curve. **C** The positive rates of serum FcγRIIa in NSCLC and healthy groups. ^***^P < 0.001. NSCLC, non-small cell lung cancer

Compared with the healthy control group (median: 1368.75 pg/mL; range: 540.92–5761.04 pg/mL, *P* < 0.001), serum FcγRIIa levels in NSCLC patients were markedly higher (median: 2282.45 pg/mL; range: 609.46–6858.44 pg/mL) (Fig. 1A). The ROC curve analysis yielded an AUC of 0.7713 (95% CI: 0.7132–0.8264), with an optimal cutoff value of 2115.88 pg/mL based on the Youden index (sensitivity: 60.06%, specificity: 86.00%) (Fig. 1B). At this cutoff, a markedly increased positive rate of 60.1% was observed for the serum level of FcγRIIa in the NSCLC set, compared to the healthy set (13%, *P* < 0.001) (Fig. 1C). Notably, 13% of healthy controls (13/100) showed FcγRIIa levels above the cutoff, indicating that elevated FcγRIIa is not entirely specific to NSCLC and may reflect baseline inflammatory status in the general population. These findings suggest the involvement of serum FcγRIIa in the NSCLC process, though its elevation may also be associated with systemic inflammatory conditions.

**Fig. 2:**
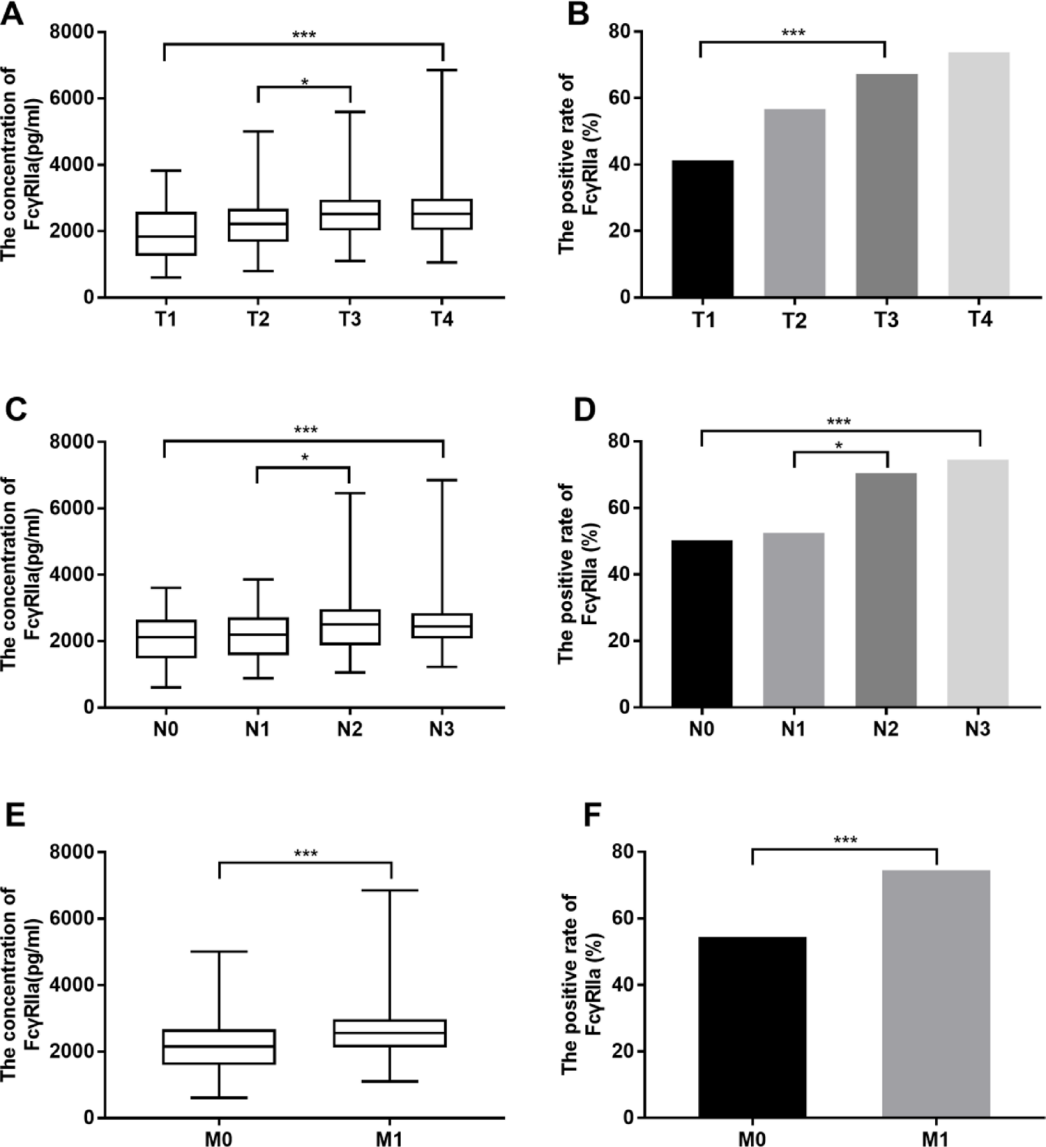
Correlations between serum FcγRIIa levels and pathologic staging of NSCLC. **A** and **B** Serum levels and positive rates of FcγRIIa changed with T-stage progression. **C** and **D** Serum levels and positive rates of FcγRIIa changed with N-stage progression. **E** and **F** Serum levels and positive rates of FcγRIIa changed with M-stage progression ^*^P < 0.05, ^***^P < 0.001. M, metastasis; N, node; NSCLC, non-small cell lung cancer; T, tumor

To disclose more correlations, further classified analyses were performed based on the pathologic TNM stages. For T-stage classification, the serum levels of FcγRIIa increased progressively from stage T1 (median: 1843.22 pg/mL, range: 609.46–3821.02 pg/mL) to stage T4 (median: 2524.18 pg/mL, range: 1057.76–6858.44 pg/mL; T1:T2, *P* = 0.023; T2:T3, *P* = 0.049; T1:T3, *P* < 0.001; T2:T4, *P* < 0.001; T1:T4, *P* < 0.001) (Fig. 2A). The positive rates showed a similar increasing trend, indicating that the serum levels of FcγRIIa are closely related to the NSCLC progression (Fig. 2B). In comparison to patients without lymph node metastasis (stage N0, median: 2115.88 pg/mL, range: 609.46–3611.26 pg/mL), the serum levels of FcγRIIa in cases with lymph node metastatic cases (stage N1-N3) rose significantly, especially in stage N2 (median: 2511.34 pg/mL, range: 1057.76–6463.52 pg/mL, *P* < 0.01) and N3 cases (median: 2440.68 pg/mL, range: 1220.08–6858.44 pg/mL, *P* < 0.001) (Fig. 2C-D). In the case of distant metastasis, the patients with stage M1 (median: 2540.06 pg/mL, range: 1057.76–6463.52 pg/mL) presented markedly elevated serum FcγRIIa levels compared to patients with stage M0 (median: 2163.27 pg/mL, range: 609.46–5009.40 pg/mL), regardless of concentration (*P* < 0.001) (Fig. 2E) or positive rate (*P* < 0.001) (Fig. 2F). All of these findings revealed a concomitant relationship between the levels of serum FcγRIIa and staging of NSCLC, suggesting the involvement of serum FcγRIIa in NSCLC progression.

### High levels of serum FcγRIIa predicted a poor outcome in NSCLC, especially in metastatic cases

**Fig. 3:**
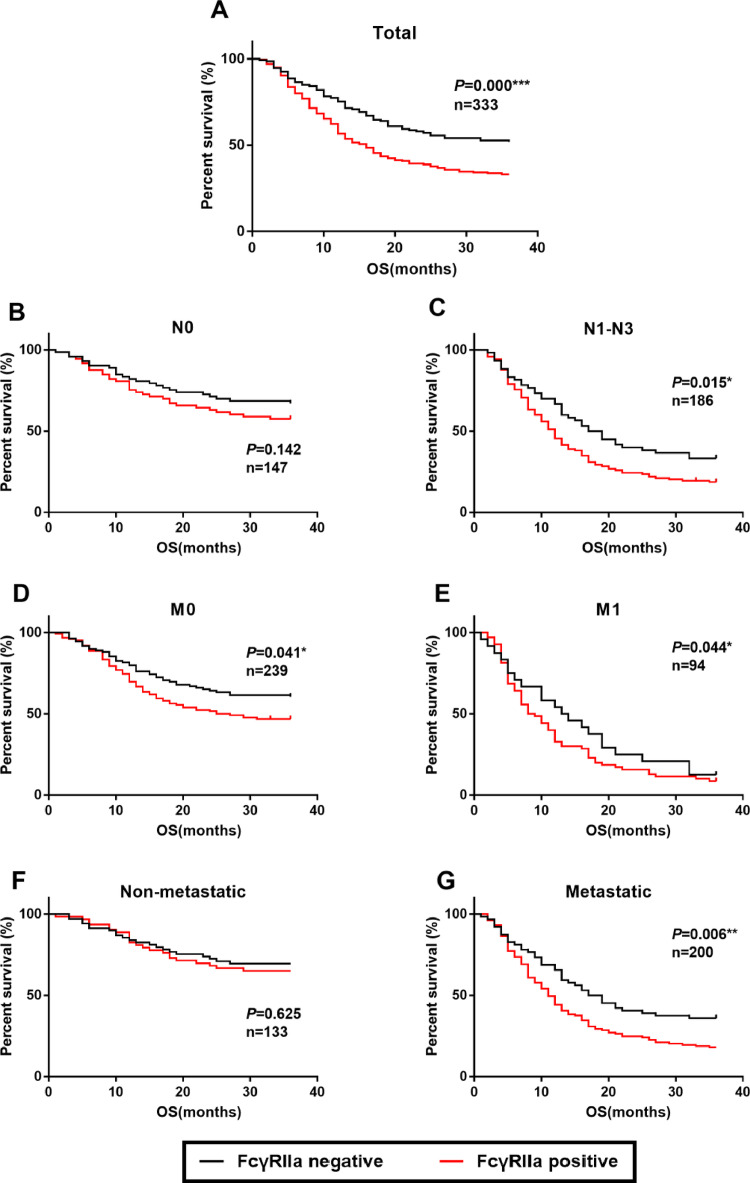
Survival outcomes of serum FcγRIIa-classified patients with NSCLC. **A** The 3-year OS was 32.5% for FcγRIIa-positive cases and 51.9% for FcγRIIa-negative cases (*n* = 333, *P* < 0.001). **B** In stage N0, the OS was 56.8% for FcγRIIa-positive cases and 67.1% for FcγRIIa-negative cases (*n* = 147, *P* = 0.142). **C** In lymphatic metastatic cases (stage N1–N3), the OS was 18.3% for FcγRIIa-positive cases and 33.3% for FcγRIIa-negative cases (*n* = 186, *P* = 0.015). **D** In stage M0, the OS was 45.3% for FcγRIIa-positive cases and 60.6% for FcγRIIa-negative cases (*n* = 239, *P* = 0.041). **E** In distant metastatic cases (stage M1), the OS was 8.6% for FcγRIIa-positive cases and 12.5% for FcγRIIa-negative cases (*n* = 94, *P* = 0.044). **F** In nonmetastatic cases, the OS was 64.1% for FcγRIIa-positive cases and 68.1% for FcγRIIa-negative cases (*n* = 133, *P* = 0.625). **G** In metastatic cases, the OS was 17.6% for FcγRIIa-positive cases and 34.4% for FcγRIIa-negative cases (*n* = 200, *P* = 0.006). ^*^P < 0.05, ^**^P < 0.01. M, metastasis; N, node; NSCLC, non-small cell lung cancer

The OS of 333 patients who underwent a 3-year follow-up examination was investigated to examine the prognostic role of serum FcγRIIa in NSCLC. Individuals with positive FcγRIIa levels had markedly shorter overall survival, with a median survival of 15 months (95% CI: 11.85–18.15), compared to patients with negative FcγRIIa levels (median: > 36 months, *P* < 0.001) (Fig. 3A). When classified by N staging, survival significance was only observed in the group of stage N1-N3, where positive cases exhibited a markedly worse median survival of 11 months (95% CI: 9.17–12.83) relative to the negative cases (median: 17 months, 95% CI: 12.66–21.34, *P* = 0.015) (Fig. 3C). In cases without lymph node metastasis (stage N0), no clear significance was observed (*P* = 0.142) (Fig. 3B), implying that serum FcγRIIa could have a more powerful prognostic potential for patients with metastatic NSCLC. This inference was further supported by the findings in the group with stage M1, where negative patients had a superior survival outcome (median: 13 months, 95% CI: 5.80–20.20), compared to positive cases (median: 8 months, 95% CI: 4.93–12.92, *P* = 0.044) (Fig. 3E). The patients were categorized into two collectives based on whether they had metastasis or not. Survival analysis was carried out to validate the aforementioned inference. In the nonmetastatic group (stage N0M0), which included cases with no lymphatic or distant metastasis, the survival curves of those positive and negative for FcγRIIa were largely similar (median: > 36 months, *P* = 0.625) (Fig. 3F). However, in the metastatic group, comprising cases with lymph node involvement (stage N1-N3) and/or distant metastasis (stage M1), FcγRIIa-negative cases exhibited a quite longer median survival of 18 months (95% CI: 11.77–22.23), compared to FcγRIIa-positive cases (median: 11 months, 95% CI: 9.10–14.88,* P* = 0.006) (Fig. 3G).

### Serum FcγRIIa was an independent prognostic indicator for patients with NSCLC, especially for metastatic cases

Cox regression models were utilized to further investigate the prognostic effects of the serum levels of FcγRIIa level and other variables in NSCLC. The outcomes of the univariate analysis revealed that the serum levels of FcγRIIa (*P* < 0.001), pathologic type (*P* = 0.021), and TNM statuses (*P* < 0.001) were distinctly related to the survival outcome of individuals (Table 2). However, variables such as age (*P* = 0.219), sex (*P* = 0.186), and smoking status (*P* = 0.273) did not show a significant correlation. Subsequent stepwise multivariate analysis revealed that only FcγRIIa levels (*P* = 0.029) and the pathologic N and M stages (*P* < 0.001) (Table 2), which directly reflect metastatic status, were independent prognostic indicators for overall survival in NSCLC patients.

**Table 2:** Cox regression analyses on the overall survival of patients with NSCLC (*n* = 333)

Variables	Risk ratio (95% CI)	*P* value
*Univariate analysis*		
Serum FcγRIIa (positive)	1.737 (1.289–2.341)	0.000^***^
Sex (female)	1.235 (0.903–1.688)	0.186
Age (> 60 years)	1.191 (0.902–1.573)	0.219
Smoking status (yes)	1.172 (0.883–1.556)	0.273
Pathologic type (S)	1.391 (1.050–1.842)	0.021^*^
pT status (T3-T4)	2.199 (1.663–2.909)	0.000^***^
pN status (N1-N3)	2.952 (2.161–4.031)	0.000^***^
pM status (M1)	2.777 (2.142–3.600)	0.000^***^
pTNM stage (Ⅲ-Ⅳ)	4.168 (3.007–5.779)	0.000^***^
*Multivariate analysis*		
Serum FcγRIIa (positive)	1.402 (1.035–1.900)	0.029^*^
N status (N1–N3)	2.241 (1.612–3.116)	0.000^***^
M status (M1)	2.068 (1.548–2.763)	0.000 ^***^

*P < 0.05, ^***^P < 0.001

*CI* confidence interval, *M* metastasis, *N* node, *S* squamous cell carcinoma, *T* tumor, *TNM* tumor-node-metastasis

Cox regression analysis was conducted to examine the connection between the levels of serum FcγRIIa and metastasis in the prognosis of both nonmetastatic and metastatic groups. In the nonmetastatic group, the majority of variables, including the levels of serum FcγRIIa (*P* = 0.623), did not show a marked effect on survival prediction for the 133 nonmetastatic cases (Table 3). The only exception was pathologic T status, which demonstrated a significant association with survival (*P* = 0.002). In contrast, the analysis of the metastatic cases revealed that the level of serum FcγRIIa (*P* < 0.001, 95.0% CI: 1.313–2.384), along with pathologic T status (*P* = 0.019, 95.0% CI: 1.063–2.012), pathologic M status (*P* < 0.001, 95.0% CI: 1.407–2.671), and TNM status (*P* < 0.001, 95.0% CI: 1.773—6.422), showed marked associations with OS. The following multivariate analysis indicated that the level of serum FcγRIIa (*P* = 0.040, 95.0% CI: 1.014–1.859), along with pathologic M status (*P* = 0.005, 95.0% CI: 1.152–2.204) and TNM status (*P* < 0.001, 95.0% CI: 2.142–4.548), exhibited marked associations with OS in patients with metastatic NSCLC. Serum FcγRIIa was identified as independent prognostic factors for patients with metastatic NSCLC.

**Table 3:** Cox regression analyses on the overall survival of patients with nonmetastatic NSCLC (stage N0M0, *n* = 133)

Variables	Risk ratio (95% CI)	*P* value
*Univariate analysis*		
Serum FcγRIIa (positive)	1.158 (0.645–2.077)	0.623
Sex (female)	0.602 (0.255–1.422)	0.247
Age (> 60 years)	1.531 (0.838–2.798)	0.166
Smoking status (yes)	0.687 (0.355–1.331)	0.266
Pathologic type (S)	0.700 (0.372–1.317)	0.268
pT status (T3–T4)	2.576 (1.408–4.712)	0.002^**^
pTNM stage (Ⅲ)	2.811 (1.187–6.655)	0.019^*^
*Multivariate analysis*		
pT status (T3–T4)	2.576 (1.408–4.712)	0.002^**^

*P < 0.05, ^**^P < 0.01

*CI* confidence interval, *S* squamous cell carcinoma, *T* tumor, *TNM* tumor-node-metastasis

## Discussion

Tumor-associated antigens, which play a role in multiple carcinogenic processes, can stimulate the host immune system through mutations, aberrant expression, and post-translational modification, leading to the production of autoantibodies. These autoantibodies form ICs that bind to Fc gamma receptors of IgG, particularly FcγRIIa (Li and Kimberly 2014). The activated FcγRIIa on inflammatory cells then mediates the downstream signaling and the release of various proinflammatory factors (Nishi et al. 2017), contributing to the establishment of tumor microenvironment that supports cancer metastasis (Altorki et al. 2019). In the present study, a serological analysis of FcγRIIa levels was performed in NSCLC patients, revealing their correlation with cancer progression and prognostic significance, particularly in cases of metastatic NSCLC. This study provides preliminary evidence for the clinical relevance of circulating FcγRIIa levels in NSCLC prognosis.

The tumor microenvironment, described as a dynamic host network and a crucial environment influencing malignant tumor progression (Spano and Zollo 2012), contains various kinds of stromal cells, including mesenchymal cells like tumor-associated fibroblasts and endothelial cell, innate cells like neutrophils and macrophages, and adaptive immune cells like B and T lymphocytes. Collectively, these various cell types collaborate to produce proinflammatory mediators, growth factors, and free radicals, which ultimately determine the fate of the tumor (Najafi et al. 2019). Substantial evidence has confirmed the critical participation of FcγRIIa in the development of IC-mediated inflammation, angiogenesis, and neoplastic progression through different cellular hosts such as neutrophils, macrophages, platelets, and so on (Tsuboi et al. 2011; Andreu et al. 2010; Miao et al. 2019). Platelet activation via FcγRIIa plays a significant role not only in platelet aggregation and thrombosis but also in IC-dependent immune response and platelet secretion (Arman and Krauel 2015). Additionally, it has been found to contribute to the prothrombotic state of malignancies, which is closely linked to cancer-associated thrombosis and metastasis (Plantureux et al. 2018; Davizon-Castillo and Di Paola 2017). Monocyte FcγRIIa also contributes to thrombotic complications through activating downstream signaling pathways and facilitating the production of tissue factors and thrombin (Tutwiler et al. 2016). Furthermore, ICs stimulate enhanced phagocytosis of macrophages by binding to FcγRIIa on apoptotic neutrophils, resulting in the discharge of proinflammatory cytokines (Gao et al. 2018) like IL-6. This cytokine, in turn, promotes the production of thrombopoietin, which is associated with cancer-related thrombocytosis (Josa et al. 2020). The results of this research emphasized the prognostic significance of the levels of serum FcγRIIa in NSCLC, primarily in cases with metastasis. Through a thorough analysis of the OS and regression models, the study revealed that the levels of serum FcγRIIa may serve as a prognostic marker in metastatic NSCLC. The survival data for classified cases, regardless of lymphatic involvement (stage N1-N3) (Fig. 3C) or distant metastasis (stage M1) (Fig. 3E) or both (Fig. 3G), suggested a potential significance of the level of serum FcγRIIa in the prognosis of metastatic NSCLC. In contrast, there was no marked connection between the levels of serum FcγRIIa and survival outcomes in nonmetastatic cases (Fig. 3B and F). Stepwise regression analyses conducted on both nonmetastatic and metastatic cases identified the levels of serum FcγRIIa as an independent predictor primarily in patients with metastatic NSCLC (Table 4) rather than in nonmetastatic cases (Table 3). The survival significance in stage M0 (*n* = 239, Fig. 3D) appeared to differ from that in other nonmetastatic groups, possibly due to a considerable number of mixed cases with lymph node metastasis (*n* = 106) in the stage M0 group, which could explain the inconsistency. Likewise, in the regression analysis of metastatic patients (Table 4), the lack of significance of pathologic lymph node metastasis (stage N1-N3, *P* = 0.546) could be explained by the presence of 14 mixed cases without lymphatic metastasis but with distant metastasis (stage N0M1). However, several limitations should be acknowledged when interpreting these findings. First, FcγRIIa is involved in general immune activation, and elevated serum levels may reflect systemic inflammation rather than NSCLC-specific processes. Furthermore, the 13% positive rate observed in healthy controls (13/100) supports this interpretation, suggesting that elevated FcγRIIa may partially reflect baseline inflammatory status rather than NSCLC-specific pathology. Unfortunately, we did not measure inflammatory biomarkers such as CRP, IL-6, or procalcitonin in this cohort. Future studies should include these markers to determine whether FcγRIIa provides prognostic information beyond that of conventional inflammatory indices. Second, this retrospective cohort only collected baseline serum samples at diagnosis, and no longitudinal measurements were available during the 36-month follow-up period. Third, treatment regimens, ECOG performance status, comorbidities, and baseline inflammatory status were not consistently documented and were therefore not included in the multivariate model. These unmeasured variables may confound the observed associations. Additionally, the present study analyzed only a single circulating marker (FcγRIIa), which limits mechanistic interpretation of the complex tumor microenvironment interactions. Future investigations should incorporate multi-omics approaches, including tumor tissue FcγRIIa expression profiling, circulating inflammatory cytokine panels (e.g., IL-6, TNF-α), and immune cell phenotyping, to elucidate the causal pathways linking FcγRIIa activation to metastatic progression and to distinguish tumor-specific effects from systemic inflammatory responses. Finally, the ROC-derived cutoff value (2115.88 pg/mL) was determined from the same cohort without external validation, and the 13% positive rate in healthy controls suggests suboptimal specificity for diagnostic purposes. These limitations underscore that FcγRIIa may serve as an additional, rather than standalone, prognostic factor in metastatic NSCLC.

**Table 4:** Cox regression analyses on the overall survival of patients with metastatic NSCLC (lymphatic and/or distant metastasis, *n* = 200)

Variables	Risk ratio (95% CI)	*P* value
*Univariate analysis*		
Serum FcγRIIa (positive)	1.769 (1.313–2.384)	< 0.001^***^
Gender (female)	1.294 (0.920–1.821)	0.139
Age (> 60 years)	1.249 (0.910–1.714)	0.168
Smoking status (yes)	1.274 (0.926–1.752)	0.137
Pathologic type (S)	1.275 (0.915–1.776)	0.151
pT status (T3-T4)	1.463 (1.063–2.012)	0.019^*^
pN status (N1-N3)	0.828 (0.448–1.530)	0.546
pM status (M1)	1.939 (1.407–2.671)	< 0.001^***^
pTNM stage (Ⅲ-Ⅳ)	3.374 (1.773–6.422)	< 0.001^***^
*Multivariate analysis*		
Serum FcγRIIa (positive)	1.373 (1.014–1.859)	0.040^*^
pM status (M1)	1.594 (1.152–2.204)	0.005^**^
pTNM stage (Ⅲ-Ⅳ)	3.121 (2.142–4.548)	< 0.001^***^

*P < 0.05, ^**^P < 0.01, ^***^P < 0.001

*CI* confidence interval, *S* squamous cell carcinoma, *T* tumor, *TNM* tumor-node-metastasis

To date, research on the clinical characteristics and significance of FcγRIIa in cancer individuals are quite limited. The majority of studies have concentrated on its significance in genetic polymorphism, which influences the anti-tumor efficacy of ADCC by interacting with IgG antibodies with differing binding affinities. It was previously observed that FcγRIIa 131H/H genotype elicits a more powerful ADCC response compared to the 131H/R heterozygous genotype in response to therapeutic monoclonal antibodies (mAbs). Hence, it was considered to have prognostic significance for patients with metastatic cancer undergoing mAb treatment (Anandappa et al. 2019; Wang et al. 2017). This notion partially aligns with the findings of the present research, indicating that the serum levels of FcγRIIa were linked to the prognosis of metastatic NSCLC cases, although the former was derived from FcγRIIa genotyping and its impact on anti-tumor responses. However, the significance of the protein level of FcγRIIa in tumor prognosis remains inadequately understood. Recent reports have indicated that patients with liver cancer exhibit stronger expression of platelet FcγRIIa compared to healthy individuals. This is accompanied by the hyperphosphorylation of downstream signaling molecules of FcγRIIa, such as Syk and PLC-γ2 (Miao et al. 2019). These molecular changes might contribute to abnormal platelet activation and the malignant progression of liver cancer. Tumor cells can also induce platelets to release angiogenic and growth factors, such as VEGF, PDGF and TGF-β (Vito et al. 2017; Hu et al. 2017). These factors facilitate tumor growth and distant metastasis by activating platelets. On the contrary, activated platelets can promote platelet aggregation and thrombosis through intercellular crosstalk with tumor cells (Jungi et al. 1988; Zara et al. 2018), contributing progressively to the metastatic cascade. The outcomes of the latest study revealed the importance of FcγRIIa in tumor progression and provided a convenient and practical approach for sampling, which may help forecast the clinical prognosis of individuals with metastatic NSCLC.

In this research, it was observed that the levels of serum FcγRIIa in patients with NSCLC increased progressively as the cancer stage progressed according to TNM staging. This suggested that the insignificance of the level of serum FcγRIIa in the prognosis of nonmetastatic cases might be attributed to lower activation of the receptor during the earlier stages of tumorigenesis and progression. However, further clinical and mechanistic investigations are required to confirm this speculation. Moreover, the prognostic significance of the level of serum FcγRIIa in patients with metastatic NSCLC could serve as an effective supplement to the early prognostic biomarker, laminin-γ2, which was previously identified (Teng et al. 2016). This expands the potential of using serum biomarkers for prognosis in NSCLC treatment.

## Conclusion

This study demonstrated that elevated serum levels of FcγRIIa were correlated with NSCLC progression. Serum FcγRIIa levels may serve as an additional, TNM stage-independent prognostic factor in patients with metastatic NSCLC. These findings should be interpreted with caution and require validation in prospective cohorts with comprehensive adjustment for treatment regimens, inflammatory markers, and comorbidities before clinical application.

## Data Availability

The datasets used and/or analyzed during the current study are available from the corresponding author on reasonable request.
